# Gouleako and Herbert Viruses in Pigs, Republic of Korea, 2013

**DOI:** 10.3201/eid2012.131742

**Published:** 2014-12

**Authors:** Hee Chun Chung, Van Giap Nguyen, Dane Goede, Chang Hoon Park, A. Reum Kim, Hyoung Joon Moon, Seong Jun Park, Hye Kwon Kim, Bong Kyun Park

**Affiliations:** Seoul National University, Seoul, Republic of Korea (H.C. Chung, C.H. Park, A.R. Kim, B.K. Park);; Vietnam National University of Agriculture, Hanoi, Vietnam (V.G. Nguyen);; University of Minnesota, St. Paul, Minnesota, USA (D. Goede);; Green Cross Veterinary Products, Yongin, Republic of Korea (H.J. Moon);; Korea Research Institute of Bioscience and Biotechnology, Daejeon, Republic of Korea (S.J. Park);; National Forensic Service, Chilgok, Republic of Korea (S.J. Park);; Institute for Basic Science, Daejeon (H.K. Kim)

**Keywords:** Bunyaviridae, Gouleako virus, Herbert virus, Republic of Korea, pigs, viruses

## Abstract

Several viruses in the family *Bunyaviridae* are pathogenic to animals and cause vector-borne zoonoses. In 2013, investigation of cause of death of 9 pigs on 1 farm in the Republic of Korea found infection with Gouleako and Herbert viruses. Subsequent investigation revealed high prevalence of these viruses among pigs throughout the country.

Several viruses in the family *Bunyaviridae*, such as severe fever thrombocytopenia syndrome virus, sandfly fever Naples virus, and La Crosse virus, cause vector-borne zoonotic problems (*1*–*7*). Recently, outbreaks of severe disease caused by Rift Valley fever virus and Schmallenberg virus produced abortion storms, resulting in a high mortality rate among newborn lambs and calves (*4*,*8*). Gouleako virus (GOLV) and Herbert virus (HEBV) have been isolated from mosquitoes (*Culex *spp*.*) trapped in Côte d'Ivoire (*9*,*10*); however, their infectivity or virulence have not been proven. Investigation of the cause of death of pigs in the Republic of Korea identified GOLV and HEBV infection.

## The Study

In March 27, 2013, a piglet, ≈8 weeks of age, on a 150-sow farm in Gyeonggi, Republic of Korea, died after onset of high fever (40°C), wasting, respiratory disease, and diarrhea. The carcass was sent to the Department of Veterinary Medicine Virology Laboratory, Seoul National University, Seoul, Republic of Korea, for diagnostics. Necropsy and microscopic examinations revealed greenish lung tissue with lymphoid depletion, consistent with severe bronchopneumonia. Despite the presence of multiple clinical signs, the results of routine tests for major pathogens in pigs (e.g., porcine reproductive and respiratory syndrome virus, porcine circovirus type 2, transmissible gastroenteritis virus, porcine epidemic diarrhea virus, *Escherichia coli, Streptococcus *spp., and *Salmonella *spp.) were negative.

To further explore cause of the death, we used the particle-associated nucleic acid –random PCR method (Technical Appendix). Sequencing and BLAST analysis (http://www.ncbi.nlm.nih.gov/blast/Blast.cgi) of the agent-specific amplicon simultaneously detected 2 viruses in lung tissue RNA samples. One partial sequence had 100% identity with 63 nt of the GOLV strain F23/CI/2004 glycoprotein gene (GenBank accession no. FJ765411). Another sequence had 97% similarity with 66 nt of the HEBV strain F23-K4 RNA-dependent RNA polymerase (*RdRp*) gene (GenBank accession no. EF423168).

Results were validated with reverse transcription PCR (RT-PCR) (Technical Appendix). We obtained partial sequences of 235 nt of GOLV and 324 nt of HEBV. These sequences had 97.1% and 96.9% similarity with GOLV and HEBV, respectively, previously isolated from mosquitoes (*9*,*10*). The sequences were registered as GenBank accession nos. KF361520 and KF361522 and designated as GOLV/P1 and HEBV/P1, respectively.

During March–May 2013, we received a total of 9 dead pigs from the same farm; they had displayed various clinical signs. We further screened these pigs for the presence of GOLV and HEBV by using the same primer sets (Technical Appendix) selective for their glycoprotein and *RdRp* genes, respectively. The results showed that the pigs were infected with GOLV and HEBV at a prevalence of 83.3% and 100%, respectively, mostly in lung samples (Table 1). The sequences obtained from this assay were registered as GenBank accession nos. KF361521 and KF361523 and designated GOLV/P8 and HEBV/P9, respectively.

**Table 1:** GOLV and HEBV screening results for dead pigs on 1 farm in Gyeonggi Province, Republic of Korea, 2013*

Pig no.	Clinical signs	Age group†	Month sample collected	Sample no.	Sample type	GOLV			HEBV		Other pathogens
						RT-PCR	qRT-PCR, copies/μL‡		RT-PCR	qRT-PCR, copies/μL‡	
1	None	Finisher	Mar	P0	Lung	–	NA		+	2.57 × 10^3^	
2	Wasting, cyanosis, fever, respiratory disorders, diarrhea	Weaned	Mar	P1	Lung§	+¶	2.03 × 10^3^		+#	1.26 × 10^2^	NA
				P2	Intestine	+	1.27 × 10^4^		+	1.16 × 10^2^	NA
3	Diarrhea, respiratory disorders	Finisher	Mar	P3	Lung	+	1.11 × 10^2^		+	1.37 × 10^3^	PRRSV, PCV2
				P4	Intestine	–	NA		–	.	
4	Diarrhea, respiratory disorders	Gilt	Mar	P5	Lung	+	2.53 × 10^3^		+	5.45 × 10^4^	PRRSV, PCV2
				P6	Intestine	–	NA		–	NA	Rotavirus, *E. coli*
5	Respiratory disorders	Weaned	Apr	P7	Lung	+	2.04 × 10^3^		+	2.78 × 10^3^	PRRSV, PCV2
				P8	Intestine	+¶	1.82 × 10^2^		–	NA	
6	Diarrhea, respiratory disorders	Grower	Apr	P9	Lung	+	5.10 ×1 0^5^		+#	5.46 × 10^3^	PRRSV
				P10	Intestine	–	NA		–	NA	
7	Diarrhea	Finisher	Mar	P11	Intestine	–	NA		–	NA	*E. coli*
8	Diarrhea	Sow	Mar	P12	Intestine	+	6.52 × 10^2^		–	NA	Rotavirus, *E. coli*
9	None	Finisher	Mar	P13	Intestine	–	NA		–	NA	NA

*E. coli, *Escherichia coli***; **GOLV, Gouleako virus; HEBV, Herbert virus; NA, not applicable; qRT-PCR, quantitative reverse transcription PCR; PCV2, porcine circovirus type 2; PRRSV, porcine reproductive and respiratory syndrome virus, Rota, rotavirus. †Samples were sorted into 6 groups: female (gilt and sow), suckling (<30 d), weaned (30–60 d), grower (60–90 d); and finisher (≥90 d). ‡Amount of virus from qRT-PCR results, copies/μL (online Technical Appendix, http://wwwnc.cdc.gov/EID/article/20/12/13-1742-Techapp1.pdf). §First sample in which virus was detected by particle-associated nucleic acid–-random PCR. ¶GenBank, GOLV (P1, KF361520; P8, KF361521). #GenBank, HEBV (P1, KF361522; P9, KF362523).

Because of the high rate of GOLV positivity, we conducted a histopathologic RNA in situ hybridization study (Technical Appendix). Hybridization signal was positive in lung and lymph node tissues and negative in intestine and control tissues. Hybridization was strong in the cytoplasm of mononuclear cells (deep blue color) (Technical Appendix Figure 1).

**Figure 1:**
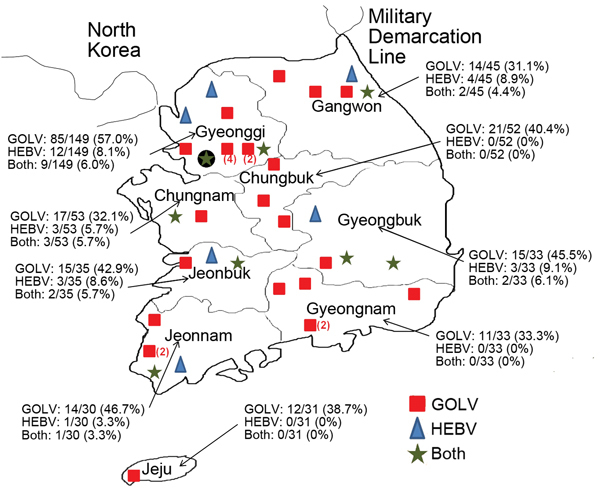
Distribution of swine farms investigated to determine cause of death of pigs, 9 provinces, Republic of Korea, 2013. The locations of farms are indicated, and the numbers and percentages of positive farms are shown in parentheses. Black dot indicates location of first case discovered. GOLV, Gouleako virus; HEBV, Herbert virus.

Using the same RT-PCR method (Technical Appendix), we investigated the prevalence of GOLV and HEBV in other swine populations in the Republic of Korea; we used the existing primer sets: (GOLV-NCF and GOLV-NCR) and (HEBV-F and HEBV-R). During March–September 2013, a total of 461 serum samples were randomly collected from 40 commercial swine farms in 9 provinces. Of these, 204 (44.3%) samples were positive for GOLV and 26 (5.6%) samples were positive for HEBV (Table 2). The rates of positivity for the investigated provinces are shown in Figure 1. When examined according to season, positive samples were more frequently found in the summer than in spring. For example, during July–August, rates were ≈65% (for GOLV) and 10% (for HEBV), but in March, rates for each virus were <10%. Rates for GOLV and HEBV positivity were higher among sows (1–4 years of age) than among pigs in other age groups (Table 2).

**Table 2:** Pig samples positive for GOLV or HEBV by RT-PCR, Republic of Korea, 2013*

Variable	No. (%) positive	
	GOLV	HEBV
Pig age group		
Gilt, n = 49	23 (46.9)	3 (6.1)
Sow, n = 76	42 (55.3)	12 (15.8)
Suckling, n = 90	20 (22.2)	5 (5.6)
Weaned, n = 90	44 (48.8)	2 (2.2)
Grower, n = 77	37 (48.1)	3 (3.9)
Finisher, n = 79	38 (48.1)	1 (1.3)
Total, n = 461	204 (44.3)	26 (5.6)

Sample collection		
Mar, n = 40	3 (7.5)	0
Apr, n = 40	5 (12.5)	1 (2.5)
May, n = 64	22 (34.4)	3 (4.7)
Jun, n = 72	27 (37.5)	3 (4.2)
Jul, n = 79	39 (49.4)	5 (6.3)
Aug, n = 82	66 (80.5)	10 (12.2)
Sep, n = 84	42 (50.0)	4 (4.8)
Total, n = 461	204 (44.3)	26 (5.6)

*GOLV, Gouleako virus; HEBV, Herbert virus; RT-PCR, reverse transcription PCR.

The study was extended to include sows on other farms in the Republic of Korea because of the major role of sows on a commercial swine farm. Pigs were divided into 3 groups and the following samples were collected: blood from healthy sows >1 year of age (n = 76), blood from abortion-problem sows (n = 13), and tissue from aborted fetuses (n = 42). Rates of virus positivity for GOLV and HEBV were higher for the 42 fetuses (33 [78.6%] and 11 [26.2%]) than for pigs in the healthy group (42 [55.3%] and 12 [15.8%]), respectively. Of the 13 abortion-problem sows, GOLV and HEBV, respectively, were found in 10 (76.9%) and 3 (23%) samples. The rates of GOLV and HEBV positivity among the healthy and abortion groups (abortion-problem sows and fetuses) were statistically compared by using the Pearson χ^2^ test in SPSS version12.0 (SPSS Inc., Chicago, IL, USA). The only significant correlation found was for HEBV infection in the abortion group; p<0.05.

Within the abortion group, pooled tissues from the 42 fetuses were screened for other pathogens (Technical Appendix). The highest rate of positivity for the fetuses was for GOLV; 17 (40.5%) of the 42 samples were positive for GOLV only. Concurrent GOLV and HEBV infection was found in 9 (21.4%) samples and GOLV and swine influenza virus in 4 (9.5%) samples; no specific pathogens were detected in 7 (16.7%) samples (Technical Appendix Table).

**Figure 2:**
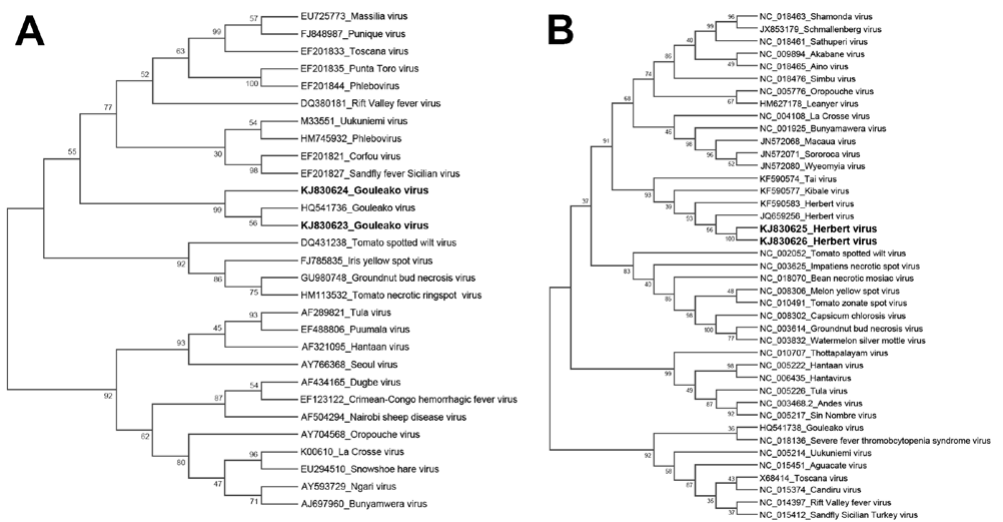
Phylogenetic analyses of Gouleako virus (GOLV) and Herbert virus (HEBV) collected from swine in the Republic of Korea, 2013 (KJ830623–J830626, in boldface), and other family *Bunyaviridae *viruses. The bootstrap consensus trees were constructed by using the maximum-likelihood method based on the general time-reversible model, implemented in MEGA version 6.06 (http://www.megasoftware.net). The phylogenetic trees for GOLV (A) and HEBV (B) were inferred on the basis of nucleotide sequences of the gene encoding nucleocapsid protein (GOLV) or RNA-dependent RNA polymerase (HEBV). The bootstrap values are shown next to the branches. *Bunyaviridae* virus sequences from previous studies (*9*,*10*) were used as reference sequences.

The phylogenetic relationships of GOLV and HEBV isolated from swine in the Republic of Korea (Technical Appendix) with the other members of family *Bunyaviridae* were analyzed; analyses were based on genes that encode the nucleocapsid protein (for GOLV) and *RdRp* (for HEBV). Of the GOLVs, the result showed that samples from the swine farms examined (KJ830623, KJ830624) clustered with GOLV strains from Africa (*9*) and showed high similarities (98.34%–98.98%) with a strain of GOLV from mosquitoes in western Africa (HQ541736) (Figure 2, panel A). Of the HEBV viruses, samples from swine farms examined (KJ830625, KJ830626) formed a branch with existing strains (KF590583, JQ659256) from mosquitoes in Côte d'Ivoire (*10*) (Figure 2, panel B). Similarities with each other were 94.46%–97.23%.

Two field GOLV strains (CP-1/2013 and CP-2/2013) used in this study were isolated from pig kidney (PK15) cells. Detailed information about the methods used to prove the results are shown in Technical Appendix Figure 2.

## Conclusions

We demonstrated that GOLV and HEBV are prevalent on swine farms in the Republic of Korea. Prevalence of these viruses was first suspected after particle-associated nucleic acid–random PCR of tissue from dead pigs, and it was proven by RT-PCR screening of a large collection of samples (serum, fetal tissue) from healthy and sick pigs throughout the country. The in situ hybridization method detected GOLV RNA in pig tissues and provided evidence in support of the presence of GOLV in the infected tissues of pigs. The findings of this study indicate that GOLV and HEBV may be associated with disease in pigs; investigation of the pathogenicity of the viruses in pigs, as well as their relation to other emerging viruses of swine, is needed.

Technical Appendix Methods used to identify Gouleako and Herbert viruses collected from swine in the Republic of Korea, 2013.
