# Supplementary material for: Gouleako and Herbert Viruses in Pigs, Republic of Korea, 2013
Source: Emerg Infect Dis. 2014 Dec;20(12):2072–5. doi: 10.3201/eid2012.131742 (PMC4257827; doi:10.3201/eid2012.131742)
Supplement: Technical Appendix — Methods used to identify Gouleako and Herbert viruses collected from swine in the Republic of Korea, 2013. [file 13-1742-Techapp-s1.pdf]

Article DOI: http://dx.doi.org/10.3201/eid2012.131742

Gouleako and Herbert Viruses in Pigs, Republic of Korea, 2013

Technical Appendix

Particle-Associated Nucleic Acid (PAN)–Random PCR

To identify the cause of the death, the particle-associated nucleic acid (PAN)-random PCR method was utilized (*1*,*2*). Porcine organs were separated into 3 groups as follows: lung tissue, intestinal tissue, and the rest of main organs including kidney, liver, lymph node, heart and spleen. It was performed with extracted DNA and RNA individually from these 3 kinds of tissues. The band from RNA/cDNA-based PAN-random PCR was purified by using the gel-extraction method and further processed for TA cloning and transformation (*2*).

RNA Extraction and RT-PCR

Total RNA was extracted by using viral RNA mini kit (QIAGEN Ltd., Manchester, UK) following the manufacturer’s instructions. The RNA was then converted into cDNA with the use of random hexamers and commercial M-MLV reverse transcriptase kit (Invitrogen, USA) following the manufacturer’s protocol. Finally, PCR reactions were performed with pathogen-specific primers using Takara Ex Taq PCR Mix kit (Takara Ltd., Korea).

The specific primers for detecting Gouleako virus (GOLV) were GOLV-F [5′-TCATTTTCAACCCCCATTGT-3′], GOLV-R [5′- CTAGCGACCTCCACACAACA-3′] which amplified 342-bp region of the glycoprotein coding gene. The specific primers for detecting Herbert virus (HEBV) were HEBV-F: [5′- TCCAACAATGACAAGCTCCA – 3′], HEBV-R: [5′- ACCATCTAGCGACCTCCAC -3′], which amplified 398-bp region of the gene encoded for *RdRp* gene.

Reconfirming Presence of GOLV

As many segments of genomic RNA of GOLV are available in GenBank, we designed another primer set targeting gene encodes for nucleocapsid protein of GOLV based on the sequence of the reference GOLV strain (EF423169). The primer pairs were GOLV-NCF [5′-TCTTGCCAGTGTGAGTTTGC-3′] and GOLV-NCR [5′-TTGGATCCAGTTGGTCTTCC-3′], which amplified 300-bp fragment of the nucleocapsid coding gene. The RT-PCR targeting nucleocapsid coding gene results confirmed the existence of this virus in the porcine samples by demonstrating the same positive results compared to the existing primer set (GOLV-F and GOLV-R).

Real-Time Quantitative Reverse Transcription PCR

The real-time quantitative reverse transcription PCR (RT-qPCR) assay using Maxima SYBR Green kit (Thermo Fisher Scientific Inc., Lithuania) was performed (*3*) to estimate viral load in tissue samples (8 positive for GOLV and 7 for HEBV). The same primer set was used (GOLV NCF and GOLV NCR) to detect GOLV. To enhance the sensitivity and accuracy, the specific primer set was designed for HEBV as follows: HEBV-QF [5′-TCAGTGGCAAATTTCCAAAA-3′] and HEBV-QR [5′-TTAAAAAGGGGGCTTCAACC-3′]. The quantity of GOLV ranged from 1.11×10^2 to 5.10×10^5 (copies/μL) and HEBV ranged from 1.16×10^2 to 5.45×10^4 (copies/μL) (Technical Appendix Table 1).

Histopathology for RNA in situ Hybridization

Dead pig no. 2 is presented in Table 1; the following 3 tissue types were collected in 10% neutral buffered formalin: lung, lymph node, and intestine. And after 1 day of fixation, samples underwent dehydration through graded alcohols and a toluene step and embedded in paraffin wax. Sections were then cut at a thickness of 5μm, mounted on "Superfrost/plus" slides (Fisher Scientific, Pittsburgh, PA, USA), and stored at room temperature. Probe sets were designed with a reference GOLV nucleocapsid protein gene (GenBank accession no. HQ541736.1, region 112–780 covered by probeset) from Affymetrix Ltd., genomics Asia teams. The in situ hybridization was utilized to QuantiGene® ViewRNA ISH tissue kit (Affymetrix Ltd., USA), according to the manufacturer’s instructions.

GOLV Isolation, Purification, and Sequencing

This study also attempted to isolate GOLV from RT-PCR positive 25 serum samples (5 pools). Both PK15, and HEK293 cells inoculated grown in Dulbecco's minimum essential medium (DMEM) containing 5% fetal bovine serum (FBS) with cultured the cells at 37°C in a 5% carbon dioxide atmosphere. The PK15 cells were detected after 42 h incubation with cytopathic effect (CPE). After observation of CPEs, the supernatant of isolates was used for further blind passage, identified by RT-PCR, real time RT-PCR, and using virus titrated using Reed and Muench method (*4*) (Technical Appendix Figure 2). After 5th passages, PK15 cell monolayers were inoculated with 10-fold diluted virus inoculums in 6-well culture plates, and incubated for 1 h. The inoculums were then removed, and the cells were overlaid with 48°C DMEM containing 1.8% agarose, 10% FBS, 1% neutral red, and incubated at 37°C in a 5% CO_2 incubator for 5 days. The formation of plaques with GOLV could be seen 100 h after infection; they appeared as round uncolored areas contrasting with the red color, were aspirated by using tips, and were suspended in DMEM. For the 5th passage, infectious virus was demonstrated by plaque assay 1.1×10^6 plaque-forming units (pfu/mL). For the clear characterization of virus isolates, the suspension of pure plaque was inoculated to PK15 cells with maintained in DMEM supplemented with 5% fetal bovine serum (FBS). After observation of CPE, the supernatant of isolates used for characterization, was identified by RT-PCR and sequencing by primer working techniques (*5*) for reference (GenBank accession no. HQ541736.1) from Macrogen Ltd., genomics teams, Korea. We obtained the complete sequences of the GOLV's S segment (1,087nt) from the 2 field GOLV strains CP-1/2013 (KJ830623) and CP-2/2013 (KJ830624).

HEBV Sequencing

Attempts were also made but failed to get HEBV isolates from 26 HEBV positive serum mentioned above. However, by another HEBV specific primers (HEBV-PF [5′-TTGAATCAGGGTTACAGGCTT-3′], and HEBV-PR [5′-GCATCGATGTCACCTTTTAGG-3′]), which amplified 1,081-bp fragment of the *RdRp* gene, we obtained partial sequences of 832bp from samples CP-3/2013 (KJ830625), and CP-4/2013 (KJ830626).

References

1. Stang A, Korn K, Wildner O, Uberla K. Characterization of virus isolates by particle-associated nucleic acid PCR. J Clin Microbiol. 2005;43:716–20. PubMed http://dx.doi.org/10.1128/JCM.43.2.716-720.2005

2. Kim HK, Park SJ, Nguyen VG, Song DS, Moon HJ, Kang BK, et al. Identification of a novel single-stranded, circular DNA virus from bovine stool. J Gen Virol. 2012;93:635–9. PubMed http://dx.doi.org/10.1099/vir.0.037838-0

3. Chung HC, Nguyen VG, Moon HJ, Kim HK, Park SJ, Lee JH, et al. Inhibition of porcine endogenous retrovirus in PK15 cell line by efficient multitargeting RNA interference. Transpl Int. 2014;27:96–105. PubMed http://dx.doi.org/10.1111/tri.12219

4. Reed LJ, Muench H. A simple method of estimating fifty per cent endpoints. Am J Hygiene. 1938;27:493–7.

5. Marklewitz M, Handrick S, Grasse W, Kurth A, Lukashev A, Drosten C, et al. Gouleako virus isolated from West African mosquitoes constitutes a proposed novel genus in the family *Bunyaviridae*. J Virol. 2011;85:9227–34. PubMed http://dx.doi.org/10.1128/JVI.00230-11

Technical Appendix Table . GOLV infections associated with abortion in pigs, South Korea, 2013

Pathogen	No. (%) samples positive
GOLV alone	17 (40.5)
GOLV + 1 pathogen	
HEBV	9 (21.4)
SIV	4 (9.5)
PCV2	2 (4.8)
PRRSV	1 (2.4)
GOLV + 2 pathogens	
HEBV, SIV	1 (2.4)
PCV2, SIV	1 (2.4)
No pathogens detected	7 (16.7)
Total	42 (100)

*GOLV, Gouleako virus; HEBV, Herbert virus; PCV2, porcine circovirus type 2; PRRSV, porcine reproductive and respiratory syndrome virus; SIV, swine influenza virus.

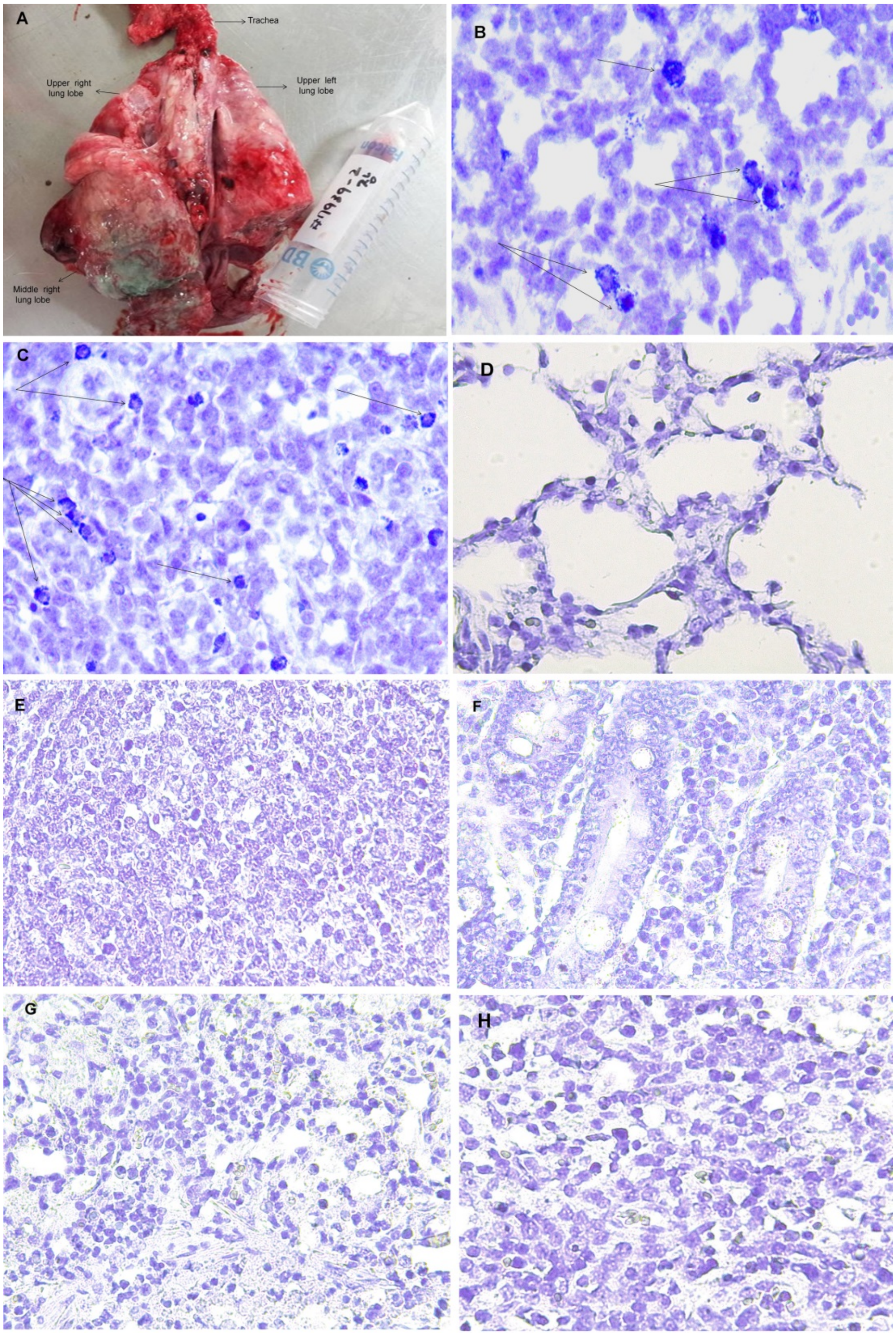

Technical Appendix Figure 1. RNA in situ hybridization assay targeting the nucleocapsid protein coding gene of Gouleako virus (GOLV) in porcine tissues. A) Gross appearance of greenish lesion in right middle lung. B) Positive signal of lung tissue and C) lymph node tissue. Negative control; tissue sections from a healthy pig (8 weeks old), which was RT-PCR negative for GOLV: D) lung, E) lymph node, F) Intestine, and omitted target probe set: G) lung, H) lymph node. The specific signal was visualized by using a standard brightfield microscope using 40× objective lens.

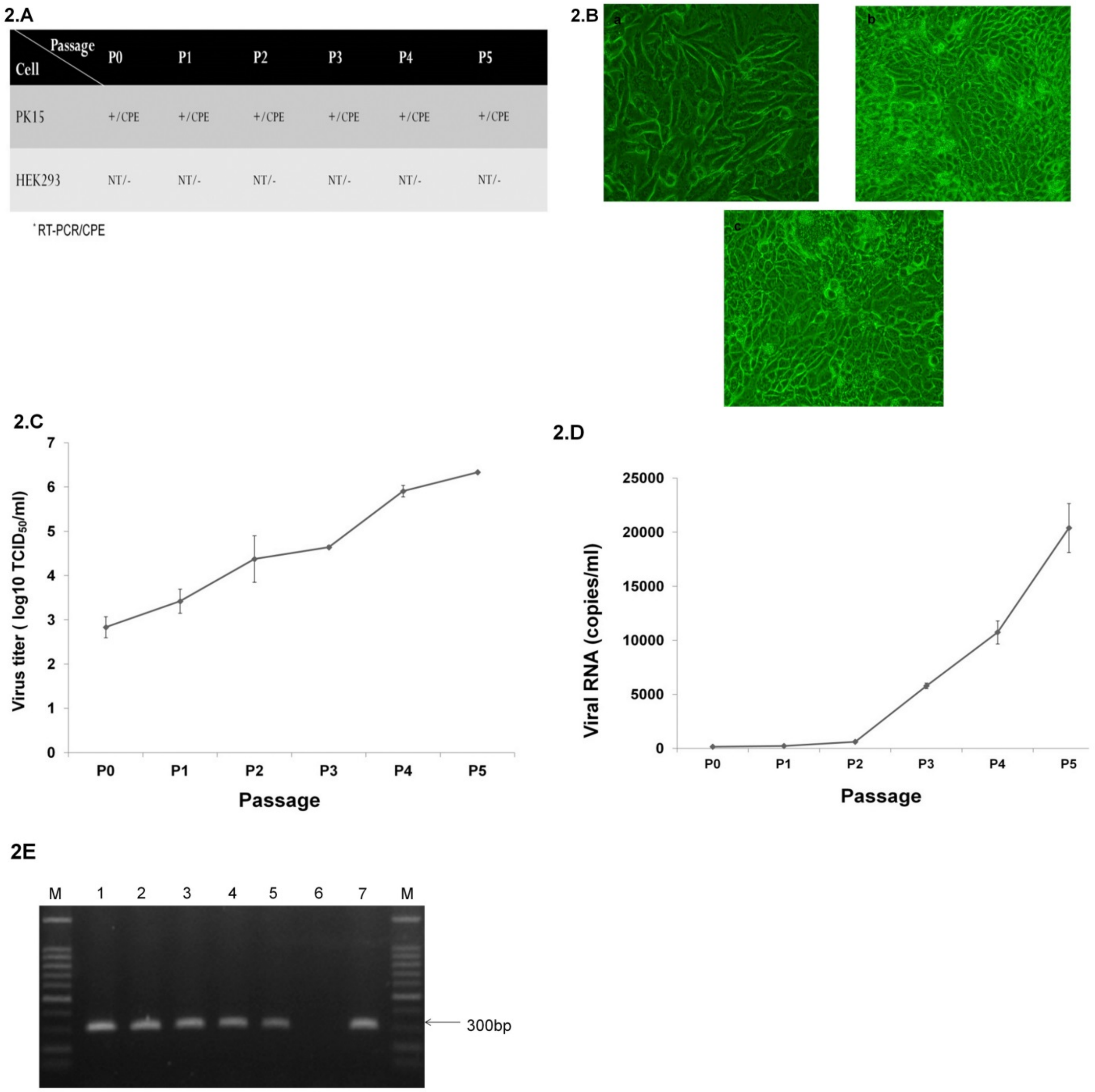

Technical Appendix Figure 2. The growth of Gouleako virus (GOLV) on pig kidney (PK15) cells. A) Isolation results of GOLV on PK15 and HEK293 cell lines. B) CPE of PK15 cells induced upon GOLV infection; a) is negative control, b) and c) show the observed CPE at the viral titer of $10^{3.615}$ $TCID_{50}$/mL and $10^{4.166}$ $TCID_{50}$/ml, respectively. C) The titers of GOLV between passages. D) The quantitative real-time RT-PCR results of GOLV between passages (reported as number of copies/mL). E) Agarose gel electrophoresis of PCR product on the nucleocapsid protein gene of GOLV. From left to right: lane M, 100-bp DNA ladder; lanes 1 to 5, passage levels 1,2,3,4, and 5, respectively ; lane 6; negative control ; lane 7; positive control.
